# Smart Characterization of Rogowski Coils by Using a Synthetized Signal

**DOI:** 10.3390/s20123359

**Published:** 2020-06-13

**Authors:** Alessandro Mingotti, Lorenzo Peretto, Roberto Tinarelli

**Affiliations:** Department of Electrical, Electronic and Information Engineering, Guglielmo Marconi Alma Mater Studiorum, University of Bologna, Viale del Risorgimento 2, 40136 Bologna, Italy; lorenzo.peretto@unibo.it (L.P.); roberto.tinarelli3@unibo.it (R.T.)

**Keywords:** rogowski coil, characterization, power quality, low-power current transformer, distorted signal, modeling

## Abstract

With the spread of new Low-Power Instrument Transformers (LPITs), it is fundamental to provide models and characterization procedures to estimate and even predict the LPITs’ behavior. In fact, distribution system operators and designers of network models are looking for all forms of information which may help the management and the control of power networks. For this purpose, the paper wants to contribute to the scientific community presenting a smart characterization procedure which easily provides sufficient information to predict the output signal of a Low-Power Current Transformer (LPCT), the Rogowski coil. The presented procedure is based on a synthetized signal applied to the Rogowski coil. Afterwards, the validity of the procedure is assessed within the Matlab environment and then by applying it on three off-the-shelf Rogowski coils. Simulations and experimental tests and results involving a variety of distorted signals in the power quality frequency range and by adopting a quite simple measurement setup demonstrated the effectiveness and the capability of the procedure to correctly estimate the output of the tested device.

## 1. Introduction

The Instrument Transformers (ITs) scenario has been radically changed after the introduction of so called Low-Power Instrument Transformers (LPITs) or sensors [1]. This new generation of transformers will facilitate the management and control of the modern power networks, which are now populated by several new actors such as Renewable Energy Sources (RES), electric vehicles, storage systems, and intelligent electronic devices. 

In terms of standards, ITs are well documented by the IEC 61869 series, in which two general documents describing the inductive ITs and the LPITs are the IEC 61869-1 [2] and -6 [3], respectively. All other documents of the series, instead, address every detail related to the operation of the transformers, from the accuracy performance to the normal and special tests that have to be performed on the ITs. Of course, the standards do not contain all possible solutions to issues that may arise when dealing with the ITs (e.g., specific accuracy tests vs. one or more influence quantities); however, the standard is evolving and changing day-by-day, including in each new version aspects that were not stressed in the previous one.

Even if ITs are not the most critical asset of the grid, such as cable joints [4,5,6] and insulators may be [7,8,9], studies on them are always clear and current. As a matter of fact, the IT is a key element that provides information on what is happening to the power network in every single instant. Furthermore, ITs are the source of information for those algorithms [10,11,12,13,14] that manage and control the power network. Hence, the quantities provided by the transformers have to be as accurate as possible in every operating condition of the network. To that end, the literature provides several works in which the accuracy of both ITs and LPITs is assessed in a variety of situations that may affect the network normal operation. For example, studies on current transformers (CTs) can be found in [15,16]. Electronic voltage transformers accuracy has been studied in [17,18], while sources of error for inductive voltage transformers (VTs) have been analyzed in [19,20]. Finally, the impact of VTs on the normal operation of the grid has been evaluated in [21,22,23].

In light of all above, this paper focuses on a particular type of Low-Power Current Transformer (LPCT), the Rogowski coil [24,25,26,27]. Thanks to its versatility, working principle, reduced dimensions, and ease of installation, in the last decade the Rogowski coil has been implemented in many applications [28] and has been subject to a variety of different studies to improve its knowledge and operation. For example, it is commonly adopted for the detection of partial discharges (PD) [29,30]. Furthermore, its large bandwidth makes it suitable for different high frequency applications [31,32,33]. Instead, in [34,35,36,37,38] the authors present several applications where the Rogowski coil is implemented to perform diagnostic and predictive maintenance measurements (for cable joints, motors, feeders, onsite calibration, etc.).

In terms of accuracy, the standard IEC 61869-10 [39] provides some guidelines to assess LPCTs, while in [40,41,42,43] their performance is evaluated for different operating conditions.

The aim of this paper is to provide a characterization procedure capable of collecting sufficient information from the Rogowski coil to estimate its output voltage when a variety of distorted test signals are injected. This is somehow related to the modeling of the Rogowski, of which the literature provides a range of works [44,45,46,47,48], but also includes omitting the information of the model and simply relying on a simplified impulse response of the device (as detailed in the next section). The developed procedure presented, which is based on a synthetized signal, can help the work of (i) network model designers, who have to include in their models all the electric assets spread among the grid; (ii) distribution system operators (DSOs) who have to deeply know how their networks operate to fully control and manage them. Therefore, the frequency range adopted in the manuscript is 50–2500 Hz, which is the power quality frequency range, in order to prove the effectiveness of the presented calibration procedure. However, a variety of new synthetized signals might be developed for whatever desired frequency range. 

Afterwards, the procedure is tested within the Matlab environment to assess is applicability. Finally, three off-the-shelf Rogowski coils have been characterized with the proposed procedure to validate their effectiveness in an actual scenario. The experimental validation procedure involves a variety of distorted testing signals with a harmonic content that varies within the power frequency limits.

The remainder of the work is structured as follows. Section 2 describes the mathematic approach that supports the overall work and the smart characterization procedure. The assessment of the procedure performed by using the Matlab environment has been tackled in Section 3. The measurement setup used to perform the experimental validity tests is explained in Section 4. As for the tests and results, they are detailed in Section 5 and Section 6, respectively. Finally, the conclusion of the work and some final comments are contained in Section 7. 

## 2. Mathematical Approach

The mathematical backbone of this section is divided into two different parts. One is dedicated to the understanding of the Rogowski coil working principle and to what could be an ideal way to obtain its transfer function. A second part is then focused on using the gathered information to detail the solution for a smart characterization, namely, the aim of this work. 

### 2.1. Theoretical Background

#### 2.1.1. Rogowski Coil

**Figure 1:**
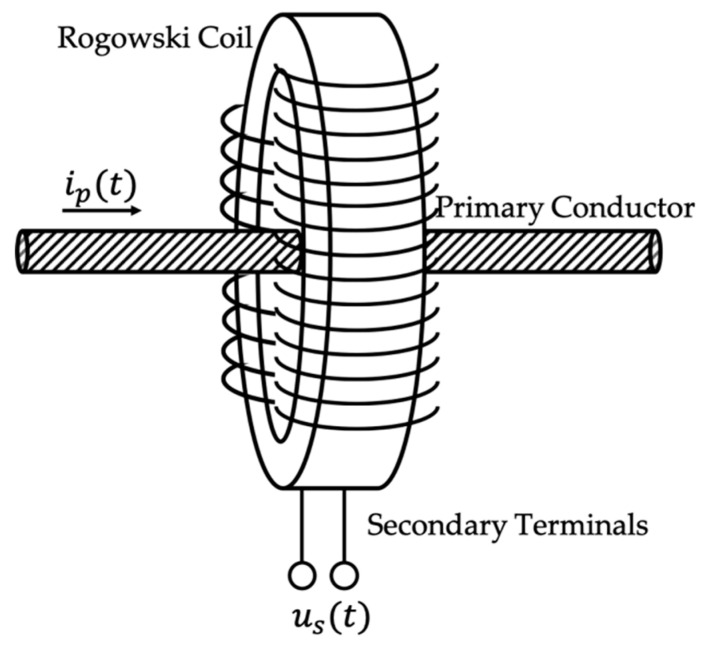
Simple Rogowski coil schematic.

The paper addresses the characterization of Rogowski coils. They can be easily recognized (see its simplified schematic in Figure 1) as a conductor wound over an insulating material (the return conductor is omitted for the sake of clarity). This aspect is what differentiate them from the inductive current transformers, wound over iron materials, and it is what guarantees their linear behavior. From Figure 1 the working principle of a Rogowski coil can be described. The primary conductor, which has current $i_{p}\left(t\right)$ has to be measured, goes inside the coil and gives an output voltage $u_{s}\left(t\right)$, described by:

(1) $$u_{s}\left(t\right)=-M\frac{di_{p}\left(t\right)}{dt}$$

From (1) it is easy to conclude that the output voltage is proportional to the derivative of the primary current and to the mutual inductance between conductors M. Furthermore, $u_{s}\left(t\right)$ is ideally 90° shifted compared to $i_{p}\left(t\right)$.

**Figure 2:**
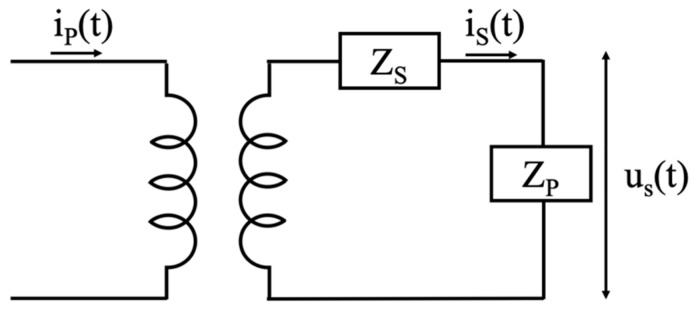
Rogowski coil equivalent circuit.

As for the equivalent circuit of the Rogowski, it is typically described, as in Figure 2, with two impedances, $Z_{S}$ and $Z_{P}.$ The former impedance consists of the series between a resistor and an inductor, whereas the latter impedance includes the parallel between a capacitor and the burden (defined in [39]). No further details on the impedances are detailed here because they are not involved in what follows and hence out of scope.

#### 2.1.2. Transfer Function

**Figure 3:**
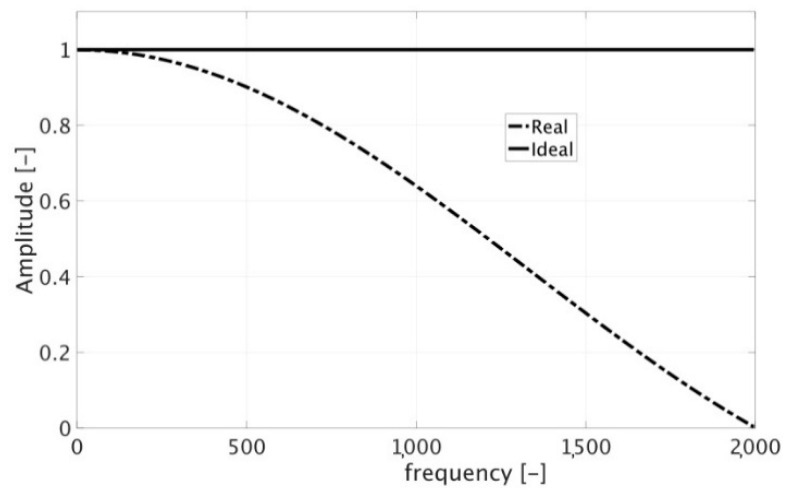
Comparison between the real (dash-dotted line) and ideal (solid line) impulse response (IR).

To deal with Rogowski coils, exploiting their linear behavior, the impulse response (IR), that is the output of a linear device when the input is an impulse, might be the easiest solution. In fact, ideally, the IR provides the transfer function of a Rogowski coil, hence, modeling it becomes straightforward. Unfortunately, the practical application of an impulse, hence obtaining the IR, is not that simple. The reason is due to the technical limitation of the typical instrumentation—in terms of resolution and capability of reproducing a real impulse—and the resulting actual IR which clearly differs from the ideal one (constant value in the frequency domain). To better clarify this aspect, note Figure 3 where the comparison between the frequency spectra of the ideal and the real IR is provided (the picture does not refer to any test, it is only a theoretical example). As a matter of fact, the spectrum of an ideal IR is a constant line which describes the same amplitude for all frequency components from minus to plus infinite (in the graph only a portion of the frequency axes is presented for obvious reasons). Turning to the actual one, it decreases after being flat for a defined range of frequencies, which depends on the instrumentation adopted to generate the impulse signal. The consequential conclusion is that, when a real impulse is applied to any device, its response should be carefully analyzed and it will differ from one frequency to another. 

### 2.2. Synthetized Signal

Considering the peculiarities of the device to be tested and the limitations of the IR test, it is possible to design and synthetize a particular signal to be used for testing the Rogowski coils. 

First of all, the frequency range that has to be tackled by the characterization process, and the frequency resolution has to be defined (depending on the application where the procedure is applied). In our case, the power quality frequency range, 50 Hz–2500 Hz, is considered together with a 50 Hz resolution (i.e., 50 harmonic components). The choice is due to the typical application of Rogowski coils to measure the current in the medium voltage distribution networks, and consequently the need for measurements that include up to the 50th harmonic [2]. 

**Figure 4:**
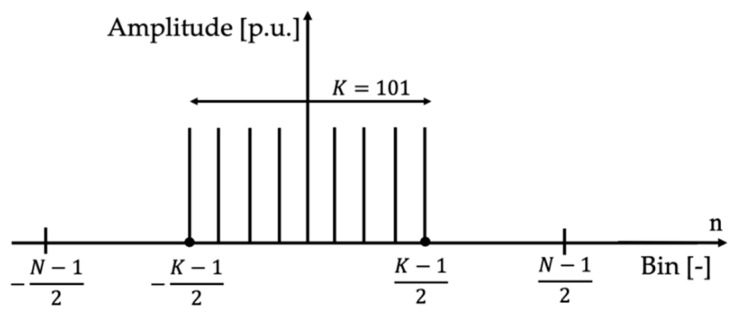
Bins in the frequency domain, representing the final test signal.

The second step is to generate a frequency domain signal, such as the one depicted in Figure 4, which reflects the previous choice. In the picture, K is the number of non-zero components and N the total number of bins (or samples) of the signal (odd in the case considered in Figure 4). 

To determine K, it should be considered as the result of the application of a Fourier Transform, which has a mean value plus one set of the desired number of harmonic components, H for example (and of course a set of mirrored, i.e., complex conjugate, negative components). Overall, *K* = 1 + *H* + *H*, and for the considered case, H=50 and hence K=101. However, the frequency domain signal can be designed for whatever range of frequency and frequency step.

**Figure 5:**
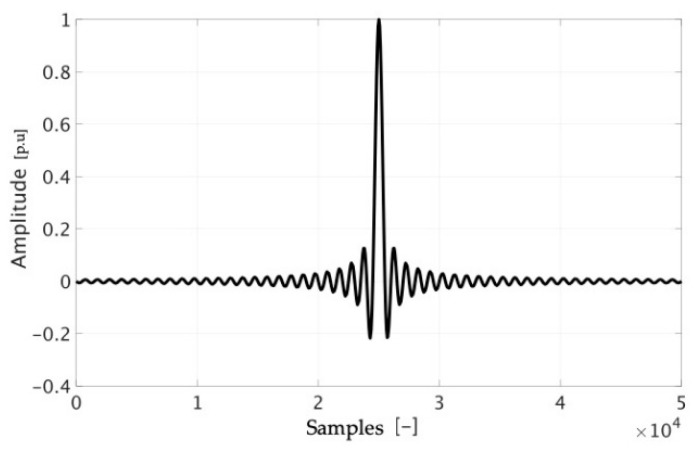
Time domain waveform, obtained from the frequency domain signal in Figure 4.

It is well-known that the signal in Figure 4 results in a sinc-shaped signal s(t) in the time domain; of course, it is a portion of a sinc, which is an infinite time domain function. Such a signal, shown in Figure 5, it is described by:

(2) $$s\left(t\right)=\frac{\sin\left(\pi nK/N\right)}{\sin\left(\pi n/N\right)}$$

where n is the variable used for the samples and introduced in Figure 4.

Consequently, the final signal to be used for the smart characterization test is s(t) and the Rogowski coil response to it may be referred to, just for simplicity, as Sinc Response (SR).

**Figure 6:**
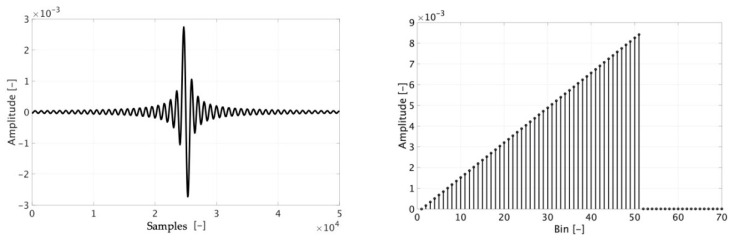
Expected time domain and frequency domain (left and right) signals obtained from the Sinc Response (SR) test applied to the Rogowski coil.

Another significant factor that can be used before performing the tests is gathered from Section 2.1.1 and, in particular, from (1). In fact, with (1) being one way of expressing the input–output relation of the Rogowski coil, it is possible to predict what the SR could be. In detail, in a qualitative way for the moment, it is possible to note in Figure 6 the expected time and frequency domain (left and right) signal resulting from SR test. Figure 6, which is just a way to understand what would be the Rogowski coil SR, has been obtained deriving s(t).

Concluding, the synthetized s(t) is going to be assessed in Section 3, by performing simulations in the Matlab environment, to understand if its application within the procedure produce satisfying results; then is used in Section 4 to perform the tests on three off-the-shelf Rogowski coils. Afterwards, the resulting SR is adopted as a simplified but more efficient IR to predict the Rogowski coils’ output voltage.

### 2.3. Smart Characterization Tests

The smart characterization introduced in this work is based on two simple tests. The first one consists of acquiring the SR of the Rogowski coil under test (RUT) (h(t)) when it is measuring the s(t) signal defined in the previous section.

The second test consists of measuring, with the RUT, a 50 Hz primary current $i_{P}\left(t\right)$ and the RUT voltage response $u_{s}\left(t\right)$ (same notation of Figure 2). The measured primary current is then convoluted with h(t) to obtain the estimated RUT’s response $u_{se}\left(t\right)$:

(3) $$u_{se}\left(t\right)=conv\left(i_{P}\left(t\right),\;h\left(t\right)\right)=\overset{+inf}{\underset{-inf}{\int }}i_{P}\left(\tau \right)h\left(t-\tau \right)d\tau$$

These two tests allow the user to completely know the behavior of his Rogowski coil. In fact, from the ratio of the rms values of $u_{se}\left(t\right)$ and $u_{s}\left(t\right)$ it is possible to find the scaling coefficient r introduced by the convolution application. In other words, to avoid the user needing to find the mathematic relation between the amplitudes of the convoluted signal and the input signals, a simple and practical test is included in the characterization procedure. Hence:

(4) $$u_{se}\left(t\right)=u_{s}\left(t\right)*r$$

Once r and h(t) are found, it is possible to apply the convolution operation to whatever $i_{P}\left(t\right)$ current, to be injected into the Rogowski coil, and estimate its voltage response.

At a glance, it is possible to note how this two-measurements calibration procedure is shorter and faster than the common characterization techniques adopted to characterize the Rogowski coils. 

## 3. Simulation in Matlab Environment

In this section the SR procedure and its validation is addressed by simulations in the Matlab environment. In particular, it is described how the Rogowski coil input–output relation, required for the simulations, has been implemented, which tests have been performed, and the related results.

### 3.1. Rogowski Coil Model Implementation

In accordance with what is presented in Section 2.1.1, the Rogowski coil behavior has been simulated implementing (1). Therefore, all test signals selected in the following are going to be computed through (1). In other words, the derivative of the test signals is going to be multiplied by a generic factor *M* to simulated the Rogowski coil response (the choice of *M* does not affect the procedure validation). The choice of adopting such a simple, but meaningful, input–output relation of the Rogowski has been taken to test the effectiveness of the proposed characterization procedure. 

### 3.2. Test Descritpion

To assess the proposed procedure a list of distorted signals has been selected and generated. In Table 1 such signals and their harmonic content are listed. As can be seen, test *#a1* is a simple 50 Hz signal for a preliminary evaluation. Afterwards, tests *#b1* to *#g2* include the 50 Hz component $I_{n}$ plus a variety of harmonic content from the 2nd to the 25th harmonic. Such a choice has been made to be aligned with the Standards IEC 519 [49] and 50160 [50]. However, the aim of these tests is to prove the validity of the characterization procedure in a variety of harmonic content of the signals.

All waveforms *#a1*–*#g2* simulates the primary currents to be measured by the Rogowski. Therefore, they have been (i) convoluted with the signal h(t) to obtain the estimated Rogowski coil output voltage and (ii) they have derived according to (1), to obtain the “real” response of the Rogowski coil. It is then from the comparison of these two results that is possible to appreciate the applicability of the smart characterization.

### 3.3. Results and Comments

To assess at a glance the applicability of the procedure, we have chosen to evaluate the results with the composite error, a well-known time domain based index which allows for appreciating the differences between two signals. It is defined, in the discrete domain and implementing the above defined variables, as:

(5) $$\varepsilon_{D}=\frac{\sqrt{\frac{1}{N}\sum_{1}^{N}{\left(ru_{s}\left(n\right)-u_{se}\left(n\right)\right)}^{2}}}{U_{s}}*100$$

where $U_{s}$ is the rms value of $u_{s}\left(n\right)$, and N is the number of considered samples. Note that the composite error is a severe index because it reflects the amplitude and phase variation between two signals. Therefore, for each of the tests in Table 1 $\varepsilon_{D}$ has been calculated and listed in Table 2. 

**Figure 7:**
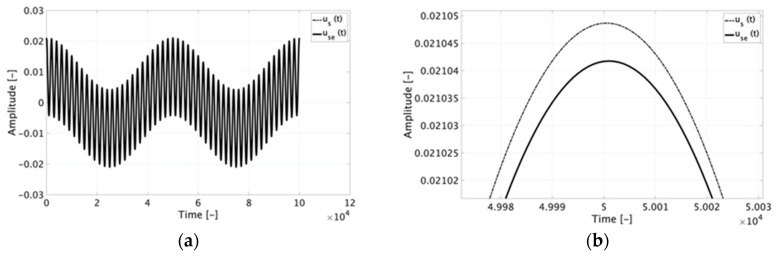
Time domain graph of test *#d1* (**a**) and a zoomed portion of it (**b**).

As can be seen in the table, all $\varepsilon_{D}$ values remain within 0.1% with the exception of tests *#e1* and *#e2* that are below 0.16%. Even in those two cases, it is not possible to distinguish the time domain waveform of the estimated and “real” signals. This is confirmed looking at Figure 7 where $u_{se}\left(t\right)$ and $u_{s}\left(t\right)$ are plotted for test *#d1* (Figure 7a). However, the slight difference between the two signals is only appreciable in the zoomed portion of graph in Figure 7b. The reason why those two tests present higher values of $\varepsilon_{D}$ may be attributed to the presence of high harmonic components compared to the others signals. In fact, it emerges from Table 2 that (i) $\varepsilon_{D}$ slightly increases for signals with high order harmonics; (ii) and $\varepsilon_{D}$ increases when the harmonic percentage with respect to the 50 Hz signal increases (comparison between tests 1 and 3 for each letter, e.g., *#b1* and *#b3*).

From Table 2 and Figure 7 it is possible to conclude that the smart characterization presented in Section 2 is fully applicable and effective, in a simulating environment, to estimate and hence predict the Rogowski coil behavior with all distorted signals tested.

In light of the results, the purpose of the next section is to assess the effectiveness of the smart characterization with experimental tests and by using off-the-shelf Rogowski coils.

## 4. Measurement Setup

**Figure 8:**
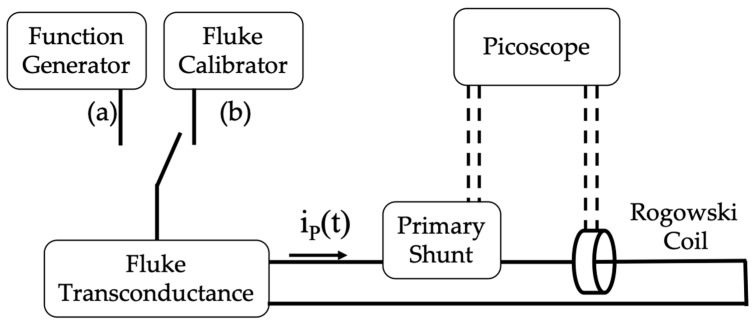
Measurement setup adopted for the two different tests, SR test using configuration (**a**), main tests performed with configuration (**b**).

Two measurement setups have been adopted to perform the tests. Both are depicted in Figure 8, wherein configurations (a) and (b) can be distinguished.

### 4.1. Sinc Response Measurement Setup

Configuration (a) in Figure 8 has been used to apply the developed signal s(t) to obtain the SR of the RUT. The measurement setup consists of:

- Function Generator (FG) Keysight 33220A. It allows for generating arbitrary waveforms and it features a sampling frequency of 50 MSa/s, frequency resolution of 1 μHz, and a frequency accuracy of ±(20 ppm + 3 pHz).
- Transconductance Fluke 52120A. Used to transduce the voltage of the FG into a current. Its main accuracy features are listed in Table 3.
- A primary shunt, 1 mΩ resistor, to measure the current generated by the transconductance and injected into the RUT. The uncertainty associated with the shunt is 0.01%. Before all tests, the shunt has been characterized in the power quality frequency range selected in this work. The results confirm the stability of the shunt in all ranges of frequency, with measured variations in the order of $2\times 10^{-6}$ Ω.
- The RUTs. Three off-the-shelf Rogowski coils have been selected to be tested. They are distinguished as *A*, *B*, and *C* for the sake of privacy and because the calibration procedure is independent of the Rogowski type. Their features are collected in Table 4. Note that the three RUTs are slightly different one from another, hence the testing sample for the procedure is not limited to only one type of device. Finally, the number of turns of *A*, *B*, and *C*, is not provided because (i) the manufacturers do not give such a characteristic; (ii) it is information that is not required to perform the presented tests.
- 16-bit PicoScope 5442D. Used to collect both outputs from the RUTs and from the shunt via BNC cables. The oscilloscope features 4 channels, 200 MHz bandwidth, sample rate up to 62.5 MSa/s at 16-bit resolution, input ranges ±10 mV to ±20 V, and gain accuracy of ±0.5% of the signal.

In brief, the synthetized signal s(t) that has been reproduced has a current signal with the system FG plus transconductance to be measured by the primary shunt and the RUT. Afterwards the voltage signals of these two devices have been collected by the PicoScope for further analysis. 

### 4.2. Main Measurement Setup

Configuration (b) of Figure 8 has been used for the experimental validation tests. What differs from configuration (a) is the use of the Fluke Calibrator 6105A instead of the FG. The choice easily set the harmonic content of the list of test signals *#b1* to *#g2*. Some of the relevant Fluke characteristics are listed in Table 5; as for the accuracy, the limits specified in Table 3 apply.

Overall, the described measurement setup can be considered a quite simple and typically adopted one. In fact, when the need is to inject distorted currents, the choice is mainly focused on transconductance amplifiers or high-power function generators depending on the desired current amplitudes.

## 5. Experimental Tests

### 5.1. Characterization Tests

The smart characterization introduced in Section 2.3 has been performed with the measurement setup described in the previous section. The first test, the SR with configuration (a), has been performed fixing a sampling frequency of 1 MSa/s and an acquiring window of 20 ms. The 20 ms window has been extracted from an acquisition of 1 s to ensure the stability of the acquired signal. As for the amplitude, the peak of s(t) has been fixed, for all RUTs, at 100 A.

The second test, which is the 50 Hz current acquisition, has been performed with a sampling frequency of 1 MSa/s, using the configuration (b) of the measurement setup, acquiring 10 periods of the signal extracted from a 1 s window. Afterwards, the r coefficients (see Equation (4)) have been computed for the three RUTs ($r_{A}$, $r_{B}$, and $r_{C}$, respectively).

For all tests (above mentioned and in what follows), the Picoscope bandwidth has been limited at its allowed minimum, 20 MHz; moreover, a 20 kHz digital filter has been set to further reduce the noise affecting the measurements.

### 5.2. Validation Tests

To experimentally validate the authors’ hypothesis that the presented characterization procedure can be used to estimate the output voltage of a Rogowski coil, a set of 17 tests has been performed (distinguished from *#a1* to *#g2*). Such tests are the same used to assess the procedure in the Matlab environment in order to have a direct comparison. Among the tests, each letter corresponds to a different harmonic content, while the number indicates a different amplitude of the harmonic compared to the 50 Hz component $I_{n}$. All tests from *#a1* to *#g2* have been performed with $I_{n}=75\;A$, a sampling frequency of 1 MSa/s and acquiring 10 periods of the signals from a 1 s window. 

Summarizing, all signals from *#a1* to *#g2* have been reproduced with the configuration *b)* of the measurement setup in Figure 8. Then, both primary and secondary signals of the RUTs have been collected; the primary has been used for the secondary estimate, the secondary, for the final comparison. The estimate of the RUTs’ responses to signals from *#a1* to *#g2* has been performed by applying (3) as detailed above.

## 6. Experimental Results and Discussion

### 6.1. Characterization Results

**Figure 9:**
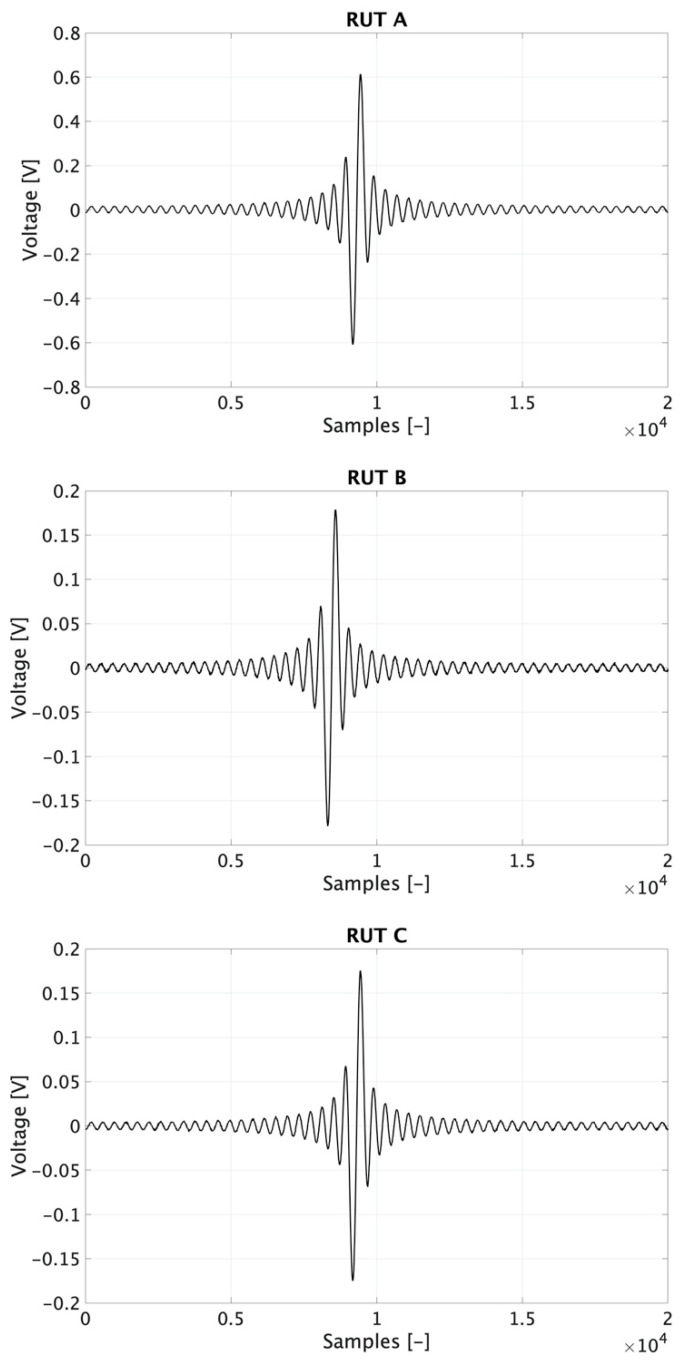
SR of RUTs A, B, and C.

The SR results, after the injection of s(t) to the three RUTs, are depicted in Figure 9. As can be seen, the three graphs confirm that (i) the three generic RUTs have very similar responses to s(t), (ii) the hypothesis made in Section 2 regarding the Rogowski coil behavior is correct. However, even if not visible from the picture, according to each RUT manufacture, the SR could be more or less affected by the presence of noise. This effect is very slightly evident in RUT *B*.

Once the SR has been obtained, the results of the 50 Hz current injection into the RUTs can be used to determine r for each RUT. In accordance with (4), the values for $r_{A}$, $r_{B}$, and $r_{C}$ are 15.7595, 15.8091, and 13.1676, respectively. The coefficients can then be used to scale the convolution results of each test defined in Section 5.2.

### 6.2. Results of the Validation Tests

As described in Section 3.3, all results are presented by using the composite error index (see (Equation (5))). Therefore, in Table 6, for each test signal *#a1* to *#g2* and for each RUT, the $\varepsilon_{D}$ values are listed. It is the result of the comparison between the estimated signal, by implementing the characterization procedure, and the RUTs voltage response measured during the experimental tests. In the table, three significant digits have been used to represent the results considering their absolute value and that they are expressed as a percentage.

Several comments can be formulated from the table considering that (i) each RUT has a different response vs. noise, vs. harmonics; (ii) each RUT comes from a different manufacturer, hence the technology implemented varies from one to the other; (iii) the distortion included in the test signals is quite vary, hence it properly stresses the RUT.

A general comment is that, looking at all RUTs, the results are quite promising considering that none of the results exceed 4.5%. This means that neither in amplitude nor in phase the estimate of the signals is never higher than that percentage. A second general comment is that, on average, the $\varepsilon_{D}$ values for RUT B are higher than the other two. However, this is perfectly in line with the higher noise dependence of RUT B, as detailed above.

A third and last general comment is aimed at confirming what obtained during the simulation tests: the higher the distortion of the primary signal, the higher is the $\varepsilon_{D}$ value. As an example, for tests #c1 to #c3 of RUT A, which involve a 7th harmonic of percentages 10, 6, and 3% compared to the 50 Hz component, the $\varepsilon_{D}$ values are 2.77, 1.95, and 1.20%, respectively. This example can be extended to all RUTs and to all test signals.

**Figure 10:**
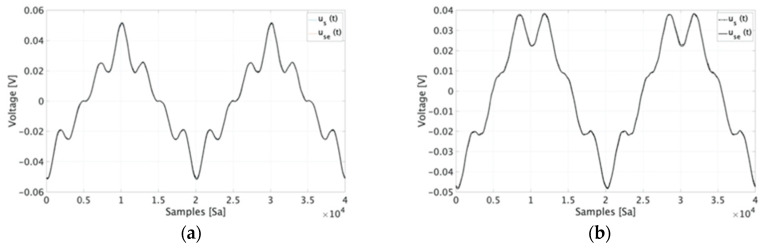

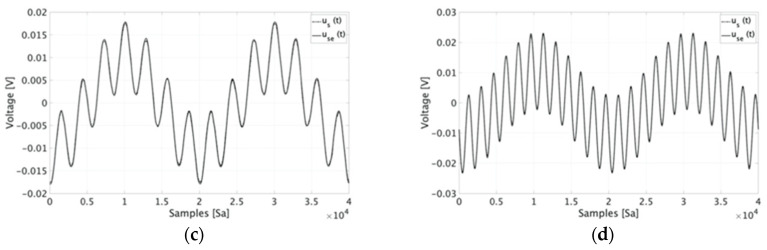
Comparison graph of the test *#a1* results for RUT A.

For the sake of completeness, in Figure 10 for time domain, graphs of the results have been presented. In particular, they refer to (a) test *#f1*, (b) test *#g1* (for RUT A), and (c) test *#c1*, (d) test *#d1* (for RUT B). As appears clear from the picture, the estimate of the secondary signal is almost undistinguishable from the reference measured signal. 

Moving to the frequency domain, the percentage variation between the 50 Hz component of the measured and estimated signals are 0.08%, 0.01%, 0.98%, and 0.02%, for the cases (a), (b), (c), and (d) of Figure 10, respectively. As for the harmonic components, the cases (c) and (d) in Figure 10 involve only one harmonic component, hence their computed percentage variation compared to the measured one are 3.07% and 2.55%, respectively. Such values confirm the effectiveness of the proposed characterization procedure.

**Figure 11:**
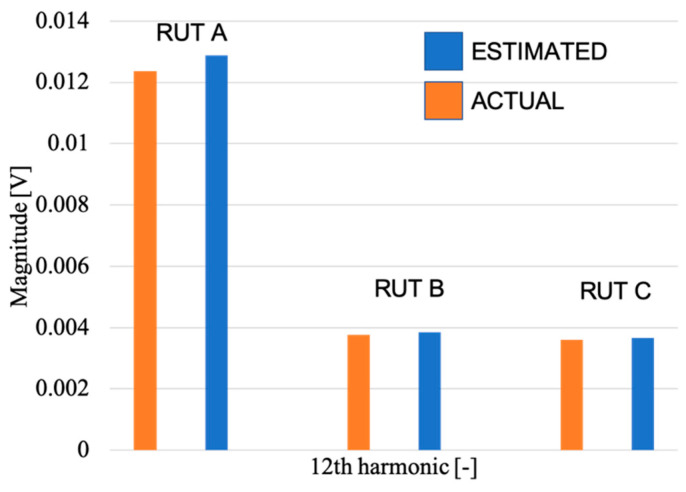
Graph with the comparison between the estimated and the actual magnitude of the 12th harmonic included in case #d3, for the three RUTs.

Finally, it is interesting to compare, in the frequency domain, the estimate of a harmonic component among the three RUTs. Such a comparison is shown in the graph of Figure 11 where the 12th harmonic of case #d3 is compared among the RUTs. From the graph the goodness of the estimation obtained applying the characterization procedure is clear.

In light of the presented results it is possible to conclude that the experimental results confirm what was obtained in the simulation tests. It is worth highlighting the benefits introduced by synthetized sin signal; in fact, it acts as a filter outside the selected bandwidth allowing the consequential reconstruction of the signal from the convolution operation. 

On the other hand, the real conditions introduce variables that are not considered in the simulating environment. For example, (i) the noise introduced from the RUTs and/or by the acquisition system; (ii) the uncertainties introduced by the measurement chain. However, from the presented results it has been confirmed that, even including the real aspects of an in-laboratory measurement, the proposed characterization procedure is effective and fully applicable. It is worth commenting on the measurement setup adopted. In fact, to implement the proposed characterization a very simple and common equipment has been deployed. Furthermore, it is fully consistent (and even simpler, due to the considered range of frequencies) with the one used to perform the sweep frequency test.

Overall, results are comforting from different points of view. First of all, the simulation tests are validated from the experimental results. In other words, the smart characterization procedure has been proven to be effective. Hence, with a faster characterization procedure than the well-known sweep frequency test it is possible to obtain the transfer function of the RUT. Second, in light of the previous conclusion and of the simplicity of the characterization developed, it is worth furthering study in this direction. In fact, the aim of the following studies, now that it has been proved the characterization validity, will be to understand its capabilities and limits extending the range of experimental/simulating studies and the number of Rogowski coils tested. 

## 7. Conclusions

The paper introduces a new smart characterization procedure for Rogowski coils. The process consists of injecting a synthetized signal in the primary windings of the Rogowski and of measuring the secondary voltage. With such a response, it is possible to estimate the secondary response of a variety of primary distorted currents. The application of this characterization aims at helping in-field and laboratory operators, to simplify and accelerate their tests by using a typical and very simple measurement setup. 

In the paper, the smart characterization has been firstly described from a mathematical perspective; afterwards, it has been tested with the Matlab environment. Finally, the characterization has been applied on three off-the-shelf Rogowski coils, testing its applicability using a variety of distorted primary signals. Both simulative and experimental results confirm the validity of the presented characterization and stimulate further research on the topic.

In fact, the possibility of estimating the behavior of low-power current transformers is a task highly required by the power network model designers and distributed system operators.

## Figures and Tables

**Table 1:** List of tests including the 50 Hz component plus harmonic content.

Test	Harmonic Content	Test	Harmonic Content
#a1	50 Hz	#d3	12th, 3% of $I_{n}$
#b1	3rd, 10% of $I_{n}$	#e1	25th, 10% of $I_{n}$
#b2	3rd, 6% of $I_{n}$	#e2	25th, 6% of $I_{n}$
#b3	3rd, 3% of $I_{n}$	#e3	25th, 3% of $I_{n}$
#c1	7th, 10% of $I_{n}$	#f1	3rd, 5th,7th, all 6% of $I_{n}$
#c2	7th, 6% of $I_{n}$	#f2	3rd, 5th,7th, all 3% of $I_{n}$
#c3	7th, 3% of $I_{n}$	#g1	2nd, 4th,6th, all 6% of $I_{n}$
#d1	12th, 10% of $I_{n}$	#g2	2nd, 4th,6th, all 3% of $I_{n}$
#d2	12th, 6% of $I_{n}$		

**Table 2:** Composite error value for each test of Table 1.

Test	$\varepsilon_{D}\;[\%]$	Test	$\varepsilon_{D}\;[\%]$
#a1	0.00632796	#d3	0.02680057
#b1	0.00813769	#e1	0.15427063
#b2	0.00707059	#e2	0.13827713
#b3	0.00652614	#e3	0.09980934
#c1	0.02587633	#f1	0.01933962
#c2	0.01808575	#f2	0.01187221
#c3	0.01099543	#g1	0.0150594
#d1	0.05940332	#g2	0.00966692
#d2	0.04536823		

**Table 3:** Accuracy characteristics of the Fluke 52120.

Current Range	% of Output	% of Range
**2**	0.015	0.070
**20**	0.015	0.060
**120**	0.015	0.020

**Table 4:** Rogowski coil under test (RUT) main features.

Feature	A	B	C
**Type**	Split-core	Split-core	Split-core
**Inner Diameter**	150 mm	115 mm	75 mm
**Accuracy**	±1%	±1%	±1%
**Ratio**	1000 A/333 mV	1000 A/100 mV	1000 A/100 mV

**Table 5:** Main characteristics of the Fluke 6105A.

Frequency Accuracy	±50 ppm
Amplitude Resolution	6 digits
Full Range Alone	20 A

**Table 6:** $\varepsilon_{D}$ values for each test of Table 1 and for each RUT.

Test	$\varepsilon_{D}\;[\%]$			Test	$\varepsilon_{D}\;[\%]$		
	RUT A	RUT B	RUT C		RUT A	RUT B	RUT C
#a1	0.752	2.57	1.89	#d3	1.64	2.76	1.82
#b1	1.024	3.01	2.49	#e1	4.58	3.27	2.36
#b2	0.814	2.79	2.12	#e2	3.76	3.18	2.17
#b3	0.782	2.73	1.95	#e3	2.46	2.74	2.15
#c1	2.77	3.03	2.20	#f1	2.17	3.13	2.06
#c2	1.95	3.17	1.91	#f2	1.26	2.71	2.37
#c3	1.20	2.93	1.76	#g1	2.01	2.75	1.95
#d1	3.47	2.65	2.21	#g2	1.25	2.79	2.32
#d2	2.71	2.73	1.89				
